# Theoretical pluralism in psychoanalytic case studies

**DOI:** 10.3389/fpsyg.2015.01466

**Published:** 2015-09-29

**Authors:** Jochem Willemsen, Shana Cornelis, Filip M. Geerardyn, Mattias Desmet, Reitske Meganck, Ruth Inslegers, Joachim M. B. D. Cauwe

**Affiliations:** ^1^Centre for Psychoanalytic Studies, University of EssexColchester, UK; ^2^Department of Psychoanalysis and Clinical Consulting, Ghent UniversityGhent, Belgium

**Keywords:** psychoanalytic schools, pluralism, single case study, single case archive, survey

## Abstract

The aim of this study is to provide an overview of the scientific activity of different psychoanalytic schools of thought in terms of the content and production of case studies published on ISI Web of Knowledge. Between March 2013 and November 2013, we contacted all case study authors included in the online archive of psychoanalytic and psychodynamic case studies (www.singlecasearchive.com) to inquire about their psychoanalytic orientation during their work with the patient. The response rate for this study was 45%. It appears that the two oldest psychoanalytic schools, Object-relations psychoanalysis and Ego psychology or “Classical psychoanalysis” dominate the literature of published case studies. However, most authors stated that they feel attached to two or more psychoanalytic schools of thought. This confirms that the theoretical pluralism in psychoanalysis stretches to the field of single case studies. The single case studies of each psychoanalytic school are described separately in terms of methodology, patient, therapist, or treatment features. We conclude that published case studies features are fairly similar across different psychoanalytic schools. The results of this study are not representative of all psychoanalytic schools, as some do not publish their work in ISI ranked journals.

## Introduction

Since Freud's discovery of the unconscious at the end of the nineteenth century, psychoanalysis has been permeated with various forms of conflict. According to its founder three factors characterize psychoanalysis; theory, clinical treatment, and research method (Freud, 1923). From its relatively short but complex history, these aspects of psychoanalysis continue to be debated by scholars both inside and outside the psychoanalytic field. According to the French philosopher Althusser (1996) heated arguments based within the field itself made psychoanalysis a schismatic discipline. Indeed, since 1910, the year Freud founded the International Psychoanalytic Association, disputes concerning theory, technique and training standards not only divided the psychoanalytic field but also led to the proliferation of different schools.

The first of these disputes concerned certain aspects of Freudian theory, promulgating the establishment of what became known as the Jungian and Adlerian schools, which were eventually no longer recognized as psychoanalytic. Shocked by these dissidences, and following a suggestion made by Jones (1955), the famous “secret committee” was formed with the aim to safeguard the theoretical foundations of Freud's theory. Whereas this well-meant initiative generated much debate, an important shift may be noted here. From the nineteen twenties onwards and regardless of how great the theoretical divergences, the so-called *schisms* (i.e., secessions in psychoanalytic associations) no longer revolved around doctrinal matters. Disputes spanned the issue of so-called lay analysis in the 19 twenties (which came down to a conflict concerning training standards—cf. Wallerstein, 1998) to those between Anna Freud and Melanie Klein in the 19 forties regarding the psychoanalytic treatment of children (concerning technique—cf. King and Steiner, 1991). These disputes were closely followed by disagreement in France concerning technique and training standards (cf. de Mijolla, 2012a,b) in the 19 fifties and early sixties. This finally led to the establishment of the Lacanian school(s).

In the second half of the twentieth century, debates have emerged on the topic of psychoanalysis as a research method (Hinshelwood, 2013). Authors from within and without the psychoanalytic field argued that the traditional case study approach advocated by Freud is not fit as a scientific tool. In this period, the interest in case studies in psychoanalysis dropped considerably, as evidenced by the relative lack in published case reports (Michels, 2000). As early as 1948, Ellis (1948) remarked that many psychoanalytic journals were moving away from publishing case studies, and sections devoted to clinical material were rarely incorporated in psychoanalytic journals. Indeed, in 1971, Anna Freud remarked, “We cannot help being conscious […] of a conspicuous […] dearth of […] complete and adequately documented case histories” (in Gardiner, 1971, p. ix). Klumpner and Frank (1991) then reviewed the 15 most cited articles in psychoanalytic journals published between 1969 and 1982 and found that not a single one of them included any significant clinical material. Indeed, after Freud, the classical lengthy case study became a rarity in psychoanalytic writing (Sealey, 2011).

Since the 19 nineties there is a renewed interest in case studies, which probably reflects the renewed interest in this method in the social sciences in general (Midgley, 2006). Indeed, since 1990, one can see a rapid increase in the number of published case studies, both in absolute numbers and relative to the total number of studies published in psychology and psychoanalysis (Desmet et al., 2013). Some authors argue that case studies are an ideal method for psychoanalysis because they allow us to grasp something of the patient's experience and access clinical material that cannot be captured in other ways (Sealey, 2011). Others consider the case study as the most suitable method to decide the theoretical debates between different psychoanalytic schools (Hinshelwood, 2013). Also outside of the field of psychoanalysis, some scholars consider case studies to be a proficient method to falsify, build and refine theories (Fluvbjerg, 2006).

Recently, an international team of researchers developed an electronic database in an attempt to organize and assemble published case studies, and systematize them according to case-descriptive information (www.singlecasearchive.com). This became known as the Single Case Archive and currently includes 446 case studies on psychoanalytic or psychodynamic treatment. In the initial process of constructing the Single Case Archive, four researchers screened all abstracts of articles published between 1955 and end of 2011 in the ISI Web of Knowledge. Search terms included “(psychoanal^*^ OR psychodynam^*^) AND (case OR vignette)” yielding 2760 hits. If decisions concerning the relevance of the publication could not be made on the basis of the abstract alone, the full article (if not available online), was sourced in libraries or ordered electronically. Case studies were selected according to the following inclusion/exclusion criteria: (1) the case study concerns individual psychoanalytic treatment, (2) the case study describes a therapy that is not only “psychoanalytically inspired” but can also be qualified as psychoanalytic in terms of therapeutic technique (e.g., music therapy, wilderness therapy, etc. were not included), (3) the case study is either the focus of the article or an illustrative vignette of sufficient size is used (i.e., more than 50% of the publication or longer than five pages), (4) the case study is written in English, French, German, or Spanish, (5) the case study is not merely a reflection on a previously published case study, but presents an original psychoanalytic treatment containing therapeutic data. The selected case studies were screened by means of a digital inventory, called the Inventory of Basic Information in Single Cases (IBISC). The IBISC was designed to assess the presence of basic information on patient (e.g., age, gender, diagnosis), therapist (e.g., age, gender, training), the actual therapy (e.g., duration, outcome, frequency) and the research method (e.g., type of data, type of analysis). The final data-matrix was used to develop an online search engine that allows users to select relatively homogeneous sets of cases (for more details see Desmet et al., 2013).

The aim of this study is to provide an overview of the scientific activity of different psychoanalytic schools of thought in terms of the content and production of case studies. To-date, no review studies on psychoanalytic case studies are available. In this study, the following research questions are explored: (1) In terms of case studies published, which psychoanalytic schools are most present in the scientific literature? (2) What are the methodology, patient, therapist, or treatment characteristics of the published case studies from the various psychoanalytic schools?

## Materials and methods

In order to gather information on the psychoanalytic orientation of each case study, all authors of a case included in the Single Case Archive were contacted by email or by post. The contact information was sourced either on the publication itself, or on the internet. First contact was made between March 2013 and August 2013. Second contact was made between September 2013 and November 2013. In order to maximize response rate, correspondence was written in English, French and/or Spanish. If the first author did not respond to our initial request, where possible, the second author was also contacted in a subsequent step. In order to stimulate authors to respond, we selected an international advisory board of psychoanalysts from different psychoanalytic schools and mentioned it in all correspondence. Ethical approval for this study was not requested as the information we collected can be considered to be part of the public domain. We contacted authors through publicly available contact details and we asked them to specify their theoretical approach as described in length in the published case study.

All respondents were made aware about precisely which case study our research questions addressed: the letters of correspondence included both the title of the case study and the actual manuscript. The purpose of our correspondence and a brief explanation of the Single Case Archive were provided, along with the following questions: (a) “*At the time you were working on this specific case, to which psychoanalytic school(s) did you feel most attached?*” Each author was given 10 options: (1) Self Psychology (1.a Theory of Heinz Kohut, 1.b Post-Kohutian Theories, 1.c Intersubjective psychoanalysis), (2) Relational psychoanalysis, (3) Interpersonal psychoanalysis, (4) Object relational psychoanalysis (4.a Theory of Melanie Klein, 4.b Theory of Donald W. Winnicott, 4.c Theory of Wilfred R. Bion, 4.d Theory of Otto F. Kernberg), (5) Ego psychology (or) “Classic psychoanalysis” (5.a Theories of Sigmund Freud, 5.b Ego psychology, 5.c Post-Ego psychology), (6) Lacanian psychoanalysis, (7) Jungian psychoanalysis, (8) National Psychological Association for Psychoanalysis (NPAP) related theory, (9) Modern psychoanalysis related to the Boston or New York Graduate School of Psychoanalysis (BGSP/NYGSP), (10) Other. Respondents could indicate one or more options; (b) “*If more than one psychoanalytic school, please list them in order of relevance. School 1: …; School 2: ….; School 3: …* ”; and (c) “*At the time you were working on this specific case, can you tell us which authors had the most influence on your work? Author 1: …; Author 2: …; Author 3: …*” The answers to question c) are not used in this study. Finally, in order to describe the characteristics of method, patient, therapist, and treatment of the case studies from the different psychoanalytic schools, we use the IBISC-ratings of the case studies.

## Results

Our first correspondence resulted in 184 responses; the second correspondence yielded 16 further responses. In total 200 responses were obtained (45% of total sample). In general, the response rate was lower for older articles: for case studies published before the year 2000, the response rate was 32, 2%, while for case studies published in the years 2006–2011, the response rate was 68, 1%.

Table 1 presents authors' responses to the first question, “At the time you were working on this specific case, to which psychoanalytic school(s) did you feel most attached?” Respondents could indicate multiple psychoanalytic schools. The results show that the two oldest schools in psychoanalysis “Object-relations psychoanalysis and Ego psychology or ‘Classic psychoanalysis’” dominate the field of published case studies. Overall, 77% of all authors (154 out of 200 respondents) reported these schools of thought to be the ones they considered themselves most affiliated with. The three more recent schools with which authors considered themselves affiliated included Self Psychology, Relational Psychoanalysis, and Interpersonal Psychoanalysis, respectively. Finally, Lacanian Psychoanalysis, Jungian Psychoanalysis, NPAP related Theory and Modern Psychoanalysis related to the BGSP/NYGSP were also mentioned as schools with which the authors considered themselves affiliated. However, as these schools were scarcely mentioned, we will not include them in the remainder of our analyses.

**Table 1:** **Affiliation to psychoanalytic schools at the time authors were working on their specific case study (multiple responses are possible)**.

**Psychoanalytic schools**	**Affiliation**	**Primary affiliation**	**Exclusive affiliation**
**Self psychology**	60	17 (28%)	5 (8%)
Theory of Heinz Kohut	18		
Post-Kohutian theories	6		
Intersubjective psychoanalysis	25		
**Relational psychoanalysis**	46	22 (48%)	10 (22%)
**Interpersonal psychoanalysis**	21	10 (48%)	3 (14%)
**Object-relations psychoanalysis**	115	54 (47%)	36 (31%)
Theory of Melanie Klein	29		
Theory of Donald W. Winnicott	45		
Theory of Wilfred R. Bion	20		
Theory of Otto F. Kernberg	23		
**Ego psychology or “Classic psychoanalysis”**	92	57 (62%)	22 (24%)
Theory of Sigmund Freud	38		
Ego psychology	20		
Post-ego psychology	23		
**Lacanian psychoanalysis**	4		
**Jungian psychoanalysis**	2		
**NPAP related theory**	1		
**Modern psychoanalysis related to the BGSP/NYGSP**	4		
**Others**, not mentioned	29		

Of all major psychoanalytic schools provided for the authors to select from, the sub-schools that most respondents deemed themselves affiliated with were as follows: within Object-relations psychoanalysis, most respondents felt attached to the theory of Donald W. Winnicott; Within Ego psychology or “Classic psychoanalysis,” most respondents felt attached to the theory of Sigmund Freud. Within Self Psychology, most respondents felt attached to Intersubjective psychoanalysis.

A considerable number of respondents (29 out of 200 respondents, or 14.5%) mentioned “Other” psychoanalytic schools. These respondents specified what they meant by “Other,” reporting names and theoretical or treatment models, including specific authors (e.g., Didier Anzieu), specific theoretical models (e.g., conflict theory), and specific treatment models (e.g., mentalization based treatment).

Of all respondents, 82 (41%) indicated that they felt attached to only one school at the time they were working on their case study; 62 respondents (31%) indicated that they felt attached to two schools; 53 respondents (26.5%) indicated three schools; 2 respondents indicated four schools; and 1 respondent indicated five schools. In other words, the majority of respondents reported feeling attached to more than one psychoanalytic school.

Where respondents reported feeling attached to several psychoanalytic schools, we asked them to list these schools in order of relevance. Below we focus on the psychoanalytic school that was deemed most relevant for respondents (where respondents reported feeling attached to only one psychoanalytic school, this school was automatically deemed the most relevant). A considerable proportion of respondents (27 out of 200) did not select one school as “most relevant.” In other words, 27 respondents who felt attached to several psychoanalytic schools considered no psychoanalytic school to be more or less relevant than others in terms of their clinical work on the case study. An overview of the responses of the other authors, who did indicate a most relevant school, is presented in Table 1 (column “Primary affiliation”). The results indicate that Object-relations theory and Ego psychology (or “Classic psychoanalysis”) were considered most relevant by most respondents. The next three schools considered relevant include Relational Psychoanalysis, Self Psychology, and Interpersonal Psychoanalysis, respectively. Interestingly, Self Psychology was less frequently selected as the most relevant by those who reported feeling attached to it. Out of 60 respondents, only 17 (28%) reported Self Psychology as the most relevant psychoanalytic school. For Relational Psychoanalysis, Interpersonal Psychoanalysis, and Object-relations Psychoanalysis, 47–48% of those who reported feeling attached to it also indicate it as having been the most relevant for their clinical work during the case study. Finally, those who feel attached to Ego psychology most frequently indicated it also as the most relevant (62%).

Finally, we analyzed how many respondents who reported feeling attached to a particular school also feel *only* connected with that particular school (see Table 1, column “Exclusive affiliation”). The highest percentage was obtained for Object-relations psychoanalysis: 36 out of 115 (31%) of those who feel attached to this psychoanalytic school, feel attached only to this school. For Ego psychology or “Classic psychoanalysis” and Relational Psychoanalysis, the percentage is lower: 24 and 22%, respectively. The lowest percentage was obtained for Self Psychology: only 8% of those who feel attached to Self psychology adhere only to this school.

For the second research question, we describe the following characteristics of case studies from the five psychoanalytic schools: methodological characteristics (type of study, year of publication), patient characteristics (gender, age, diagnostic information), therapist characteristics (gender), and treatment characteristics (completed/not completed, duration of treatment, outcome).

Overall, 12% of the case studies in the Single Case Archive are empirical case studies (i.e., cases using systematic quantitative and/or qualitative analysis) and 88% use no systematic qualitative or quantitative method (Desmet et al., 2013). Table 2 presents the percentage of empirical case studies for the different psychoanalytic schools. Although most of the published case studies used no systematic qualitative or quantitative methodology, empirical case studies have been published in all psychoanalytic schools. For all five psychoanalytic schools, the mean year of publication is 2003, which confirms the recent uplift of case study research in all psychoanalytic schools.

**Table 2:** **Methodological, patient, and therapist characteristics for case studies from the different psychoanalytic schools**.

**Psychoanalytic schools**	**% empirical studies**	**Mean year of publication**	**% Analyst female**	**% Patient female**	**Mean age patient**	**Most frequent DSM-IV category**
Self psychology	12%	2003	41%	57%	34.2	Anxiety
Relational Psychoanalysis	11%	2004	41%	59%	38.3	Mood
Interpersonal Psychoanalysis	19%	2003	30%	67%	25.9	Anxiety
Object-relations psychoanalysis	8%	2004	43%	50%	32.7	Anxiety
Ego psychology or “Classic psychoanalysis”	10%	2002	42%	54%	34.1	Anxiety

Overall, the Single Case Archive includes 46% male and 34% female therapists (in 20% of the cases, no information on the therapist's gender was available). In terms of patients 47% are male and 53% are female (Desmet et al., 2013). As can be seen in Table 2, this gender imbalance permeates case studies from all psychoanalytic schools. Among the case studies from Interpersonal Psychoanalysis, the balance is particularly uneven, with only 30% female therapists and 67% female patients. The mean age of all patients is 32.6 years old, but there is a rather large difference between the case studies from Interpersonal Psychoanalysis (25.9) and Relational Psychoanalysis (38.3). Within all five psychoanalytic schools, case studies with under age patients have been published. In the Single Case Archive, diagnostic terms were assigned (when available, i.e., 93% of 446 cases) to one of the main categories of the DSM-IV. The most frequently occurring diagnoses were anxiety disorders and mood disorders. Table 3 presents the most frequently occurring DSM-IV diagnosis for all case studies across different psychoanalytic schools. It appears that most case studies can be situated within the DSM-IV category of anxiety disorders, apart from those that use Relational Psychoanalysis.

**Table 3:** **Treatment characteristics for case studies from the different psychoanalytic schools**.

**Psychoanalytic schools**	**≥4 sessions/week**	**Treatment completed**	**Treatment duration**	**Treatment success**
Self psychology	39%	73%	48.4	71%
Relational psychoanalysis	50%	50%	54.6	61%
Interpersonal psychoanalysis	50%	79%	45.1	71%
Object-relations psychoanalysis	42%	74%	40.8	61%
Ego psychology or “Classic psychoanalysis”	44%	72%	43.8	62%

Forty-four percent of the case studies in the Single Case Archive concern a treatment with a session frequency of 4–6 times a week. There is some variation between the psychoanalytic schools, with Self Psychology having 39% of psychoanalysis, and Relational and Interpersonal Psychoanalysis both having 50% highly intensive treatments. Overall, 51% of all cases studies in the Single Case Archive concern treatments that are completed at the moment of the writing of the case (Desmet et al., 2013). As can be seen in Table 3, only 50% of case studies from Relational Psychoanalysis report completed treatments. Of all completed cases, the average duration of the treatment was 45.7 months (*SD* = 32.96; min. = 3 months; max. = 180 months). In this context, case studies from Relational Psychoanalysis stand out with an average duration of 54.6 months. In 65% of all cases, the treatment was considered successful by the author; in 35% of all cases, the treatment was considered to have either failed or produced mixed outcomes by the author. The results in Table 3 show that there is little difference between the psychoanalytic schools in this respect.

## Conclusion

The present study found that the two oldest psychoanalytic schools, Object-relations psychoanalysis and Ego psychology or “Classic psychoanalysis,” were most productive in publishing case studies on ISI-Web of Knowledge between the years 1955 and 2011. Object-relations psychoanalysis emerged as the most influential school: more than half of all authors (115 out of 200; 58%) reported feeling affiliated with Object-relations psychoanalysis, and just under half (54 out of 115; 47%) consider this school as the most relevant for them. This probably reflects the high number of independent Kleinian institutes across Europe and Latin-America. It is noteworthy that approximately one-third (31%) of those who feel attached to Object-relations psychoanalysis, feel only attached to this school. In other words, this psychoanalytic school appears to have a body of loyal practitioners who publish case studies. Ego psychology or “Classic psychoanalysis” also emerged as very influential: 92 authors reported feeling attached to this school and 57 out of 92 authors (62%) consider this school as the most relevant for their clinical work. This may reflect the hegemony of Ego psychology or “Classic psychoanalysis” in the American Psychoanalytic Association (APsaA) and the International Psychoanalytic Association (IPA), as many analysts are primarily trained in this orientation. However, it can also be noted that only 22 out of 92 (24%) of those who feel attached to Ego psychology or “Classical psychoanalysis” do not feel attached to any other psychoanalytic school. This indicates that case study authors from Ego psychology or “Classic” psychoanalysis are generally more pluralistic in comparison to Object-relations psychoanalysis.

A different picture emerges when we look at the school of Self Psychology. In a sense, this school seems to be the most successful of the three newer schools (Self Psychology, Relational Psychoanalysis, and Interpersonal Psychoanalysis) since up to 60 authors of case studies (30%) reported feeling attached to this school for their clinical work. At the same time, however, a comparatively low percentage (28%) of its adherents consider this school to be the most relevant, and a high percentage (92%) feel attached to other psychoanalytic schools as well. This finding may reflect something of the history of Self Psychology. When Kohut developed Self Psychology, he broke with the theory of Ego psychology, but did not form an independent institute (Summers, 2008). Instead, he remained in the (Ego psychological) Chicago Institute for Psychoanalysis. Until now, institutes devoted solely to Self Psychology are few in number and lack visibility. However, at the same time Self Psychology has extensive and intensive influence in many psychoanalytic programs (Summers, 2008). This influence is clear in our results, as 60 out of 200 (30%) case study authors in our sample reported that they feel attached to Self Psychology.

The publication of case studies in ISI ranked scientific journals emerged rather recently in the 19 nineties, even in the “older” schools of Object-relations psychoanalysis and Ego psychology or “Classic” psychoanalysis. In the context of this century-old discipline, this is a relatively new trend in the field, but it reflects the renewed interest in case study research in the social sciences (Midgley, 2006). Moreover, it seems to be a trend that has emerged in all psychoanalytic schools. The description of the case studies of different psychoanalytic school yielded some interesting trends. Whereas clinical case studies make up the main part of published case studies, empirical case studies have also been published within all psychoanalytic schools. Case studies in Relational Psychoanalysis stand out because they involve older patients and longer treatments. Case studies in Interpersonal Psychoanalysis tend to involve young, female patients and male therapists. Case study authors from both schools tend to report on intensive psychoanalysis in terms of session frequency.

Overall, the differences between case studies from five major psychoanalytic schools considered from the criteria maintained in this study are fairly small. This is due to the fact that there is considerable overlap between schools. The majority (118 out of 200 respondents, or 59%) of psychoanalysts and psychotherapists that participated in this study reported that they feel attached to two, three, four or five psychoanalytic schools. In fact, a non-negligible number of authors (27 out of 200 respondents, or 13.5%) were unable (or did not want) to select one out of several psychoanalytic schools as most relevant for their clinical work. In other words, they feel attached to several psychoanalytic schools and have no particular preference for one of them. Only 82 respondents (41%) adhere to only one psychoanalytic school for their clinical work. This result is much lower than expected, given the sometimes ardent disputes between analysts from different psychoanalytic schools (Green, 2005; Summers, 2008). The so-called incompatibility between different psychoanalytic theories does not appear to have inhibited the majority of participants in this study from feeling attached to more than one psychoanalytic school. Perhaps the origin of *schisms* between psychoanalytic schools is related more to technical matters and training standards than it is to doctrinal matters. Whereas certain authors may have been trained in a specific psychoanalytic institute (e.g., Ego psychology) and use the associated techniques, they appear to also make use of other psychoanalytic models to gain insight into their work. This is not to suggest that different psychoanalytic theories become integrated in the minds of such analysts. The question as to how analysts combine different theories or whether they find a common ground between different theories is not addressed in this study. However, one may hypothesize that such a combination is dictated by the clinical situation itself, with the analyst drawing from different theoretical frameworks in order to select the most appropriate treatment interventions. It is also possible that this combination is only made in an abstract way, i.e., when the analyst thinks and writes about the patient. Further research might elucidate how psychoanalysts combine and use different psychoanalytic theories when choosing interventions during treatment.

This study has several limitations. First, our review is based on one source only; the Single Case Archive. This is not an exhaustive database. It contains only case studies from ISI-ranked journals. Certain groups of psychoanalysts (e.g., Lacanian analysts) have their own circuit of journals and are thus less inclined to publish in ISI-ranked journals. Case studies from this particular school are not included in this review. Moreover, we were only able make contact with 45% of the Single Case Archive's authors, leaving us with no information on more than half of the other case studies. A third limitation concerns the self-report format of our study. As the authors commented on their psychoanalytic affiliation retrospectively, this is subject to memory biases. Moreover, a discrepancy may exist between how the authors see themselves and how they actually work clinically. It is possible that a psychoanalyst may feel most attached to one psychoanalytic school, but works clinically according to the principles and techniques of another psychoanalytic school. This limitation could be addressed with expert-judgments on the psychoanalytic affiliation of case studies. Finally, a reviewer noted that the exact wording of our question might have influenced the responses: we asked the authors to which psychoanalytic school they *feel most attached*. The question probes the emotional relation, rather than the intellectual relation or the social relation (e.g., membership) to a psychoanalytic school. The reference to attachment evokes the personal, even transferential relation psychoanalysts have toward a psychoanalytic school and it's proponents. However, respondents might think differently about this and interpret the question differently. Moreover, the naming of the psychoanalytic schools might be controversial to some respondents: “Jungian psychoanalysis” might sound as a contradiction in terms, and defining “ego psychology” as “classical psychoanalysis” could be contested. The list of psychoanalytic schools is a compromise that intends to be recognizable to every case study author.

## Author contributions

JW has done the data collection, supervised the completion of this research project and contributed to the critical revision of the manuscript. SC has done the data analysis and she contributed to the writing of the Results and Discussion Sections. FG has written the paragraphs in the introduction on the history of psychoanalysis and he established the list of psychoanalytic schools. MD has contributed to the design of the study and he has written the Results Section. RM has contributed to the design of the study and she has written the Discussion Section. RI has contributed to the design of the study and she has edited the manuscript. JC has contributed to the data analysis and contributed to the critical revision of the manuscript.

### Conflict of interest statement

The authors declare that the research was conducted in the absence of any commercial or financial relationships that could be construed as a potential conflict of interest.
